# Formation and Control of Zero‐Field Antiskyrmions in Confining Geometries

**DOI:** 10.1002/advs.202202950

**Published:** 2022-08-17

**Authors:** Licong Peng, Konstantin V. Iakoubovskii, Kosuke Karube, Yasujiro Taguchi, Yoshinori Tokura, Xiuzhen Yu

**Affiliations:** ^1^ RIKEN Center for Emergent Matter Science Wako 351‐0198 Japan; ^2^ Department of Applied Physics University of Tokyo Bunkyo‐ku 113‐8656 Japan; ^3^ Tokyo College University of Tokyo Bunkyo‐ku 113‐8656 Japan

**Keywords:** antiskyrmions, confining geometries, Lorentz TEM, spintronics

## Abstract

Magnetic skyrmions and antiskyrmions have attracted much interest owing to their topological features and spintronic functionalities. In contrast to skyrmions, the generation of antiskyrmions relies on tunning both the magnitude and direction of the external magnetic field. Here, it is reported that antiskyrmions can be efficiently created via quenching and robustly persist at zero field in the Fe_1.9_Ni_0.9_Pd_0.2_P magnet with the *S*_4_‐symmetry. It is demonstrated that well‐ordered antiskyrmions form in a square lattice in a confining micrometer‐scale square geometry, while the antiskyrmion lattice distorts in triangular, circular, or rotated‐square geometry; the distortion depends on the relative configuration between sample edges and the two *q*‐vectors arising from the anisotropic Dzyaloshinskii–Moriya interaction, in good agreement with micromagnetic simulations. It is also characterized transformations from antiskyrmions to skyrmions and nontopological bubbles at different directions and values of external field. These results demonstrate a roadmap for generating and controlling antiskyrmions in a confining geometry.

## Graphical abstract

Magnetic antiskyrmions have been investigated in noncentrosymmetric magnets. The effect of geometry on their formation and stability is crucial for understanding the intrinsic properties of antiskyrmions, yet it has rarely been studied. Here, the authors demonstrate the formation and control of confined zero‐field antiskyrmions and the impact of sample geometry on the lattice form, revealing the inter‐particle and particle‐edge interactions.

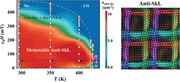

## 1 Introduction

Topologically protected spin‐whirling structures, including skyrmions and antiskyrmions, have been extensively investigated because of their emergent electromagnetic properties and potential spintronic applications.^[^ ^1^, ^2^, ^3^, ^4^, ^5^ ^]^ Chiral skyrmions, arising from the lack of inversion symmetry and consequent Dzyaloshinskii–Moriya interaction (DMI), are studied in helimagnets with chiral‐symmetry (*P*2_1_3 and *P*4_1_32) cubic structures, such as MnSi,^[^ ^2^, ^6^, ^7^ ^]^ FeGe,^[^ ^8^ ^]^ and Co‐Zn‐Mn.^[^ ^9^, ^10^, ^11^ ^]^ The skyrmion lattice (SkL) with a six‐fold symmetry can be stabilized in a narrow temperature (*T*)‐magnetic field (*µ_0_H*) window below the Curie temperature (*T*_C_).^[^ ^2^, ^6^, ^8^, ^9^, ^10^, ^12^ ^]^ Quenching the thermodynamic SkL phase, hence avoiding the first‐order transition into a conical magnetic state, enables SkL to occupy relatively large *T‐µ_0_H* regions.^[^ ^6^, ^9^, ^10^, ^12^, ^13^, ^14^ ^]^ Magnetic skyrmionic bubbles with an integer winding number originate from the magnetic dipolar interaction and hence exhibit two degrees of the helicity freedom. They can also be generated by quenching in uniaxial ferromagnets without DMI.^[^ ^15^, ^16^ ^]^ In contrast to the creation of chiral skyrmions (which is related to the isotropic DMI) and skyrmionic bubbles (related to the magnetic dipolar interaction), the formation of antiskyrmions in noncentrosymmetric magnets^[^ ^4^, ^5^, ^17^, ^18^, ^19^, ^20^, ^21^ ^]^ stems from the coexistence of anisotropic DMI and magnetic dipolar interaction.

Antiskyrmions containing alternating Bloch‐ and Néel‐type spin twists have been observed in noncentrosymmetric magnets with *D*_2d_^[^ ^4^, ^5^, ^19^, ^20^ ^]^ and *S*_4_^[^ ^21^, ^22^ ^]^ symmetries. The anisotropic DMI is essential for the formation of the opposite helicity of the helix and hence antiskyrmions, while the magnetic dipole interaction is important for the square shape of antiskyrmions as it favors Bloch‐type twists and suppresses the Néel‐type parts (Bloch lines).^[^ ^5^, ^19^, ^20^, ^21^, ^23^ ^]^ Tuning the external magnetic field, temperature, and sample geometry induces transformations among the antiskyrmions, elliptical skyrmions, and non‐topological bubbles via the creation, propagation, and annihilation of Bloch line pairs.^[^ ^5^, ^21^ ^]^ To create antiskyrmion lattice at room temperature (RT), previous studies^[^ ^5^, ^21^, ^22^, ^24^ ^]^ has to vary the temperature as well as both the magnitude and direction of the applied magnetic field. Only sparse antiskyrmions can be created by applying the magnetic field normally to the thin plate because the ferromagnetic state with a stripe domain structure is thermodynamically stable at RT. The oblique fields with tilt angles ranging from 10° to 40° **off** the normal direction alter the free‐energy landscape and yield a lattice of non‐topological bubbles.^[^ ^5^, ^21^, ^24^ ^]^ Such bubbles then transform into antiskyrmions when the oblique field is set back to the normal direction. These observations hint to a demand for an efficient way to control the antiskyrmions and their stability, not only for applications, but also for understanding the intrinsic properties of antiskyrmions.

One possible tool to address the stability of antiskyrmions is their interactions with sample edges in confining geometries.^[^ ^25^, ^26^ ^]^ Antiskyrmions tend to collapse into half‐antiskyrmion‐like textures near the edges in Heusler compounds with *D*_2d_ symmetry.^[^ ^25^ ^]^ On the contrary, chiral skyrmions deform^[^ ^27^ ^]^ or annihilate^[^ ^28^ ^]^ at the sample edges in helimagnets with a *P*2_1_3‐symmetry cubic structure. The different behaviors near the sample edges are possibly caused by the DMI differences, which originate from dissimilar crystal symmetries in those two magnetic systems: the wave vectors of anisotropic DMI‐induced modulated spin textures are fixed and well aligned orthogonally to the crystalline orientations in antiskyrmion‐hosting systems,^[^ ^5^, ^19^, ^21^, ^25^, ^26^ ^]^ while the wave vectors of helix arising from isotropic DM vectors in chiral skyrmion‐hosting helimagnets are relatively flexible.^[^ ^13^, ^27^ ^]^

**Figure 1:**
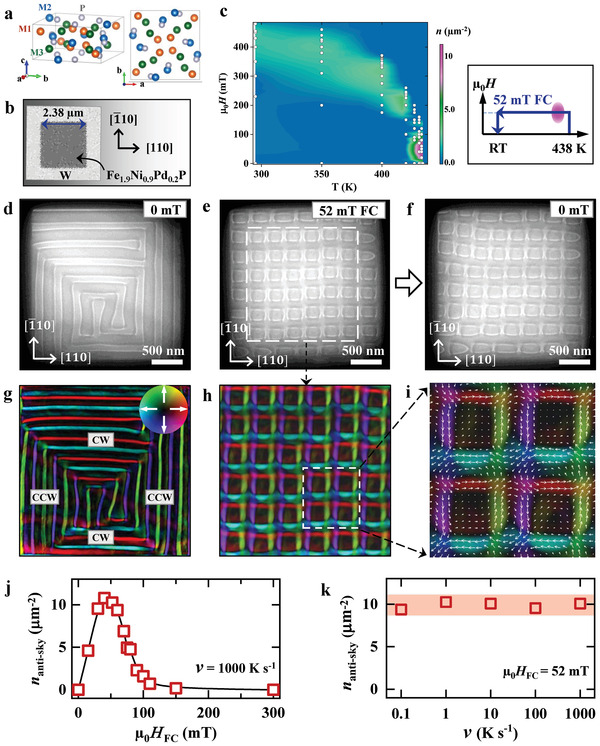
Formation of metastable antiskyrmion square lattice in a square geometry. a) The crystal structure of noncentrosymmetric tetragonal magnet M_3_P with *S*_4_ symmetry. The *a*‐*b* plane projection is shown at the right. Blue, green, and orange spheres represent three crystallographically inequivalent metal sites (M1, M2, M3) that are shared by Fe, Ni, and Pd, whereas the gray spheres correspond to the P element.^[^ ^21^, ^22^ ^]^ b) A scanning electron microscopy image of a Fe_1.9_Ni_0.9_Pd_0.2_P thin plate, in which the border with tungsten W defines a square geometry. The sample edges are aligned to the [110] and [$\bar{1}$10] crystal axes. c) Thermodynamically stable phase diagram of magnetic textures in the square‐geometry Fe_1.9_Ni_0.9_Pd_0.2_P thin plate as determined during the field increase after zero‐field cooling from above *T*_C_ to the respective measurement temperature. The right panel of (c) outlines the 52‐mT field‐cooling (FC) process with the magnetic field and temperature passing through the equilibrium phase (pink ellipse). d–f) Under‐focus L‐TEM images of (d) the helical stripes at 0 mT, the metastable antiskyrmion lattice at 52‐mT field (e), and at zero field (f) at room temperature (RT) after a 52‐mT FC from 438 K to RT. g–i) Magnetic induction field maps of (g) the helical stripes with clockwise (CW) and counter‐clockwise (CCW) helicities derived from (d), and (h, i) the antiskyrmion lattice outlined by the dashed white squares in (e) and (h), respectively. j‐k) Density of antiskyrmions *n*_anti‐sky_ as functions of (j) the magnitude of cooling field µ_0_ *H*_FC_ at a cooling rate *v* = 1000 K s^–1^, and (k) the cooling rate *v* at µ_0_ *H*_FC_ = 52 mT. The color wheel in (g) denotes the in‐plane direction of the magnetic component, and the darkness denotes the out‐of‐plane component.

In this study, we have demonstrated the generation and control of confined antiskyrmions in the noncentrosymmetric magnet Fe_1.9_Ni_0.9_Pd_0.2_P with a tetragonal structure and *S*_4_ symmetry [**Figure** **1**a]. The antiskyrmions, their lattice form, and stability are systematically studied in several samples with various geometries, such as a square, circle, and equilateral triangle, by Lorentz transmission electron microscopy (L‐TEM) combined with micromagnetic simulations.

## 2 Results and Discussions

We fabricated a micrometer‐sized thin plate of Fe_1.9_Ni_0.9_Pd_0.2_P with a square shape in a dimension of 2.38 µm × 2.38 µm × 0.16 µm [see Figure 1b and Figure S1, Supporting Information]. The sample edges are aligned to the [110] and [$\bar{1}$10] crystal axes. The dark borders in L‐TEM images originate from the tungsten deposited onto the sample edges (Figure S1, Supporting Information). The helices with two orthogonal wavevectors parallel to [110] and [$\bar{1}$10] axes appear at 0 mT (see Figure 1d), and are well aligned to the square borders. The clockwise (CW) helicity for horizontal stripes and counter‐clockwise (CCW) helicity for vertical stripes are revealed at RT by the corresponding magnetic induction field map (Figure 1g). The field direction is illustrated using a hue‐saturation‐lightness color wheel in Figure 1g. We then examine the magnetic spin textures by varying the external magnetic field at respective temperatures after zero‐field cooling, and summarize a *T*‐µ_0_ *H* phase diagram in Figure 1c. We observe dense magnetic objects like antiskyrmions at temperatures near *T*_C_ (Figure S2, Supporting Information), schematically shown as the pink area on the right panel of Figure 1c. They are most likely thermodynamically stable.

We then apply a 52‐mT field cooling (FC) procedure (the right panel of Figure 1c) with the magnetic field and temperature passing through the thermodynamic equilibrium phase (pink ellipse), and carry out L‐TEM observation at RT. This process can largely extend the temperature‐magnetic field stable region of antiskyrmions, which can persist at zero field, in contrast to the previous studies^[^ ^21^, ^22^, ^29^ ^]^ where the antiskyrmions were formed at a high field. Metastable antiskyrmions are generated at RT after FC at 52 mT (Figure 1e). Except for several non‐topological bubbles around sample edges, a square lattice of antiskyrmions is observed in the sample with square geometry. The corresponding magnetic induction field map is shown in Figure 1h. Enlargement of four antiskyrmions (Figure 1i) demonstrates that the Bloch‐type spin spirals with opposite helicities arising from the anisotropic DMI are parallel to the [110] and [$\bar{1}$10] axes, which are also parallel to sample edges, while the Bloch lines (Néel‐type twists with divergence of magnetization) are located at the four corners of square antiskyrmions. Importantly, the magnetic induction fields near the Bloch lines bind the neighboring antiskyrmions together, and hence promote the formation of a robust square lattice of antiskyrmions. The decrease of magnetic field induces expansion of antiskyrmions observed at 0 mT in Figure 1f. Simultaneously, some antiskyrmions near the sample edges collapse into half‐antiskyrmion textures (at the left edge or near the upper left corner). Both phenomena affect the distortion of the square lattice of antiskyrmions.

Figures 1j,k presents the density of antiskyrmions *n*_anti‐sky_ (the number of antiskyrmions per square micrometer) as a function of the magnitude of cooling field µ_0_ *H*_FC_ and cooling rate *v*, respectively. The *n*_anti‐sky_ peaks at µ_0_ *H*_FC_ ≈ 50 mT (Figure 1j), showing an optimal value of the cooling field for quenching antiskyrmions in the present setup (Figure S3, Supporting Information). Figure 1k demonstrates almost the same *n*_anti‐sky_ for *v* ranging from 0.1 to 1000 K s^–1^ (see L‐TEM images in Figure S4, Supporting Information), revealing that *v* does not significantly affect the formation of metastable antiskyrmions. Here the cooling rate is controlled by a rapid heat flow through a micro‐electromechanical‐system‐based chip (E‐chip mounted on the TEM sample holder, Fusion Select, Protochips, USA).^[^ ^30^ ^]^ The *n*_anti‐sky_ – *v* relation in the present material makes a marked contrast with that for the metastable skyrmion formation in the chiral magnet MnSi, which requires *v* ≥ 10^2^ K s^–1^ to avoid the thermodynamically stable conical phase,^[^ ^6^, ^12^ ^]^ while it is similar to that for metastable skyrmions formation observed in Co‐Zn‐Mn compounds with chemical disorder.^[^ ^9^ ^]^

**Figure 2:**
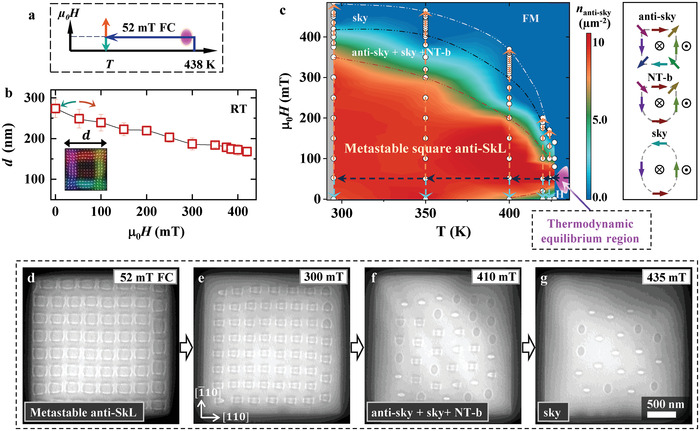
State diagram of the metastable antiskyrmion lattice. a) Schematic of the field cooling process: the sample is cooled from *T* = 438 K (>*T_C_*) to the desired temperature while keeping the magnetic field at 52 mT; then the field strength is decreased to zero or increased. b) Plot of the antiskyrmion size *d* ( defined as shown in the inset) versus magnetic field at 295 K. The error bars in (b) denote the averaged size of antiskyrmions, including the deformed ones located around sample edges. c) State diagram of the quenched metastable antiskyrmion lattice (anti‐SkL) derived from L‐TEM observations. The lattice was prepared by cooling the sample into the thermodynamically stable phase (colored in pink) (see details in Figure S2, Supporting Information). The color bar in (c) indicates the density of antiskyrmions *n*_anti‐sky_. H and FM in (c) denote helical stripes and ferromagnetic phase, respectively. Right panel of (c) outlines the spin textures of an antiskyrmion (anti‐sky), a nontopological bubble (NT‐b), and a skyrmion (sky). d–g) Under‐focus L‐TEM images showing the transition from (d‐e) the metastable anti‐SkL into (g) skyrmions (sky) at 435 mT via (f) a mixed state of anti‐sky, sky, and NT‐b at 410 mT.

We then investigate the state diagram of quenched antiskyrmions by sweeping the external magnetic field at various temperatures (the experimental procedure is illustrated in **Figure** **2**a). Figure 2b shows the field dependence of the antiskyrmion size at RT, revealing that antiskyrmions expand at low fields and gradually shrink with an increasing magnetic field to gain Zeeman energy. Figure 2c summarizes the *T*‐*µ_0_H* state diagram of antiskyrmions derived from systematic L‐TEM observations (Figures 2d‐g): the metastable antiskyrmions are obtained by quenching the narrow thermodynamic equilibrium phase (pink region in Figures 1c and 2c) to temperatures far below *T*_C_, and hence the antiskyrmion lattice state is expanded to a relatively large *T*‐*µ_0_H* phase space, including RT and zero field (red region in Figure 2c).

At a relatively weak cooling field of 52 mT, L‐TEM observation (Figure 2d) demonstrates that the close‐packed antiskyrmions develop over the whole sample area extending to the edges. However, in a relatively high field of 300 mT, antiskyrmions move away from the edges and gather around the sample center (Figure 2e), implying that the force between the antiskyrmions and the sample edges is repulsive, while keeping the topologic textures intact. A similar behavior was reported experimentally and explained theoretically for chiral skyrmions in helimagnets.^[^ ^31^, ^32^, ^33^, ^34^, ^35^, ^36^, ^37^, ^38^ ^]^

When we increase the field to 410 mT, the antiskyrmion lattice transforms into a mixture of antiskyrmions, non‐topological bubbles, and elliptical skyrmions (Figure 2f). A lattice composed of elliptical skyrmions with opposite helicities can be generated by further increasing the field up to 435 mT (see Figure 2g and the corresponding magnetic induction map in Figure S5, Supporting Information). The skyrmion formation under a high magnetic field is attributed to the strong magnetic dipolar interaction, which has been clarified in our previous reports.^[^ ^21^, ^22^ ^]^ Note here that antiskyrmions start to transform or annihilate near the sample edges, possibly due to the inhomogeneous demagnetization field around the edges.^[^ ^39^ ^]^ The total number of various magnetic textures in the course of transformation in Figures 2d‐g is not preserved and decreases with increasing magnetic field. When the magnetic field is increased further, the skyrmion lattice forms regular polygons, wherein the number of skyrmions *n_sky_* can be adjusted with a single‐particle precision (Figure S6, Supporting Information).

**Figure 3:**
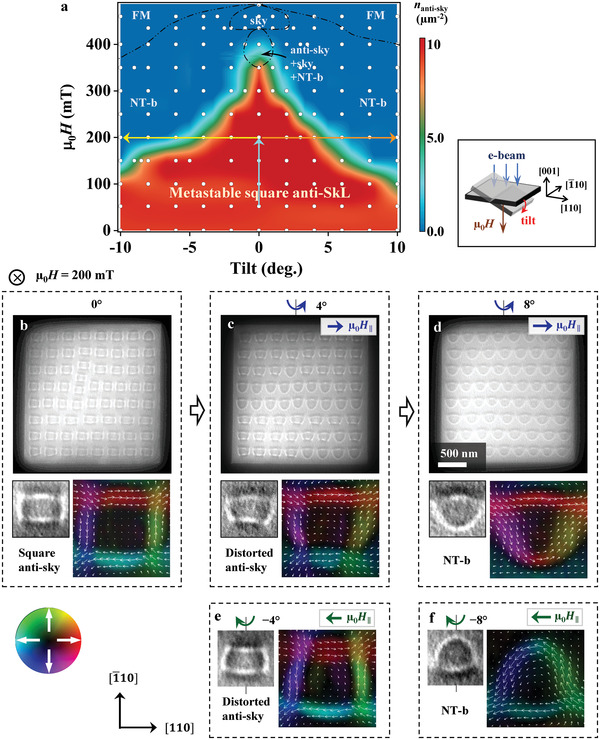
Transformation of metastable antiskyrmions in a tilted field. a) A tilt angle‐magnetic field state diagram of the metastable antiskyrmions created by FC at 52 mT. A schematic of the tilt‐experiment configuration is shown in the right panel of (a). The sample plate is tilted around the [$\bar{1}$10] axis from the plane perpendicular to the electron beam and the field. The magnetic field direction is antiparallel to the [001] axis and parallel to the wavevector of incoming electron beam. The lines with arrows in (a) exemplify the experimental procedure: we first adjust the field strength to a certain value such as 200 mT at zero tilt; then we tilt the sample and hence induce an in‐plane field µ_0_ *H_||_*. b–d) L‐TEM images showing the transformation of antiskyrmions upon sample tilt: (b) antiskyrmions in a square lattice at 0° tilt and a normal field of 200 mT, (c) a mixture of antiskyrmions and nontopological bubbles at a 4° tilt, and (d) a triangular lattice of non‐topological bubbles at an 8° tilt. The bottom panels in (b‐d) show the L‐TEM images and corresponding magnetic induction field maps of an individual (b) square‐shaped antiskyrmion, (c) trapezoid‐shaped antiskyrmion, distorted by the in‐plane field component resulting from the 4° tilt, and (d) non‐topological bubble created at the 8° tilt. e‐f) L‐TEM images and corresponding magnetic induction field maps of an individual (e) distorted antiskyrmion at −4° and (f) nontopological bubble at −8° (Figure S7, Supporting Information).

Next, we have studied the collapse dynamics of metastable antiskyrmions by tilting the sample at RT from the plane perpendicular to the applied magnetic field and incoming electron beam, which is schematically drawn in the right panel of **Figure** **3**a. The experimental procedure, denoted by the lines with arrows in Figure 3a, is as follows: the initial state is a metastable antiskyrmion lattice created by FC at 52 mT and zero tilt; we then increase the field to 200 mT while keeping zero tilt, and then tilt the sample to +10° or −10°. The L‐TEM image and the corresponding magnetic induction field map in Figure 3b show a square lattice of square‐shaped antiskyrmions at a normal field of 200 mT. When we tilt the sample by 4° around the [$\bar{1}$10] axis and hence induce an in‐plane magnetic field *μ*_0_ *H*_∥_, antiskyrmions distort to a trapezoid shape (Figure 3c) via the displacement of Bloch line positions while keeping the topological number intact (and some of the antiskyrmions are already turned into non‐topological bubbles as a result of annihilation of a Bloch‐line pair). Therein, the bottom Bloch domain wall (blue color with left‐pointing white arrows in the induction field map of Figure 3c) becomes shorter, while the opposite one (red color with right‐pointing white arrows) is longer. A similar but opposite deformation of antiskyrmions appears when the sample is tilted by −4° (Figure 3e and Figure S7, Supporting Information): the upper Bloch domain wall (red color with right‐pointing white arrows in Figure 3e) becomes shorter. When the tilt angle is increased to 8° (Figure 3d) or −8° (Figure 3f and Figure S7, Supporting Information), all antiskyrmions collapse into non‐topological bubbles, accompanied by a change of the topological number from 1 to 0.

We repeated the sample‐tilting experiments at different magnetic field values, and summarized the results in Figure 3a as a contour plot of the density of antiskyrmions. Below 100 mT, the antiskyrmions are robust against tilting in the range −10° to +10° (i.e., in‐plane field ranging from −17 to +17 mT), while they collapse into non‐topological bubbles upon tilting as the applied field is increased. For example, at a relatively high field of 300 mT, antiskyrmions persist only in a narrow range of ±1° [*μ*_0_ *H*_∥_ ≈ ±5 mT], revealing the sensitivity of antiskyrmions to the oblique magnetic field.

**Figure 4:**
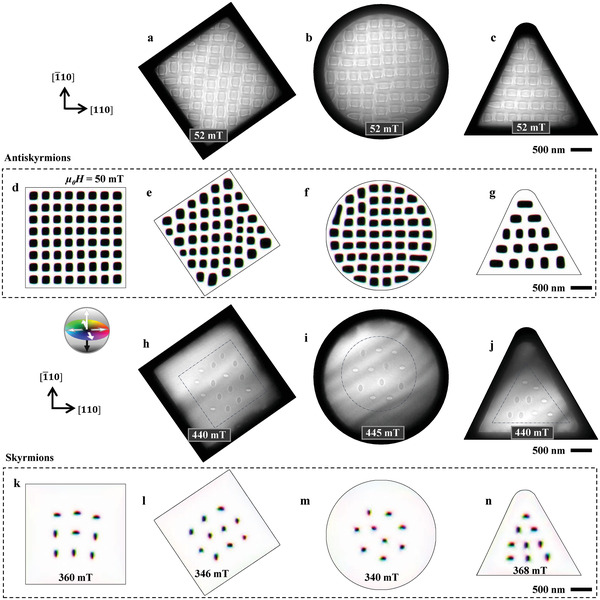
Geometry effect on the metastable antiskyrmion/skyrmion lattice. a–c, h–j) L‐TEM images of (a‐c) antiskyrmion lattices at 52 mT, and (h‐j) the skyrmion lattices generated at 440 mT (h), 445 mT (i), and 440 mT (j) in samples with different geometries of (a, h) a rotated square, (b, i) a circle, and (c, j) a triangle. d–g, k–n) Micromagnetic simulations of (d‐g) antiskyrmion lattice relaxed from a random magnetization state at 50 mT, and (k‐n) skyrmions generated at the specified values of magnetic field.

The antiskyrmion stability is affected by sample edges,^[^ ^25^ ^]^ yet the geometry effects on the antiskyrmion lattice form have not been investigated. Hence in this work, we have combined L‐TEM observation with micromagnetic simulations (see Methods) to demonstrate the stability of confined antiskyrmions, in a square sample with edges rotated by 34° or 124° away from the ***q*** direction (parallel to the [110] direction) (**Figure** **4**a), in a circular sample (Figure 4b), and in a triangular sample (Figure 4c). The helical stripes exhibit fixed ***q***‐vectors (Figure S8, Supporting Information) which are determined by the intrinsic DMI, hence they hardly depend on the sample geometry. Figures 4a–c show the L‐TEM images of the metastable antiskyrmion lattice at 52 mT and RT in the three selected samples. A large number of irregular magnetic objects are observed near the sample edges, such as nontopological bubbles and half‐antiskyrmion‐like textures. The square antiskyrmion lattice is highly distorted, especially near the sample edges, yet it persists around the sample center. Particularly, antiskyrmions form a triangular configuration in the sample with triangular geometry (Figure 4c). The lattice distortion arises from the misalignment between the sample edges and the two orthogonal ***q***‐vectors of the anisotropic helices. The experimental results agree qualitatively with micromagnetic simulations (Figures 4d–g), where exchange interaction, anisotropic DMI, magnetic dipolar interaction, and uniaxial anisotropy are taken into account by using Mumax3^[^ ^40^ ^]^ (see details in Methods). The anisotropic DMI vectors are defined parallel to [110] and [$\bar{1}$10] directions, or the square edges of Figure 4d so as to match the experimental observations. In simulations, antiskyrmions exhibit square shapes in a well‐ordered square lattice in Figure 4d, while they deform near sample edges accompanied by the lattice distortion when the edges misalign with the two ***q***‐vectors in Figures 4e–g, manifesting the interaction between the metastable antiskyrmions and sample edges at 50 mT.

With increasing the magnetic field, the antiskyrmion lattices transform into skyrmion lattices (Figures 4h–j). The skyrmions gather around the sample center in relatively high magnetic fields due to the repulsion of skyrmions by the sample edges.^[^ ^32^ ^]^ In addition, skyrmions are confined in areas with different forms, as indicated with dashed lines in Figures 4h–j upon changing the sample geometry: skyrmions reside in a rotated‐square area in the rotated‐square sample (Figure 4h), in a circular area in the circle sample (Figure 4i), and in a triangular area in the triangular sample (Figure 4j), exemplifying again the repulsive interaction between the skyrmion and the sample edge at this relatively large magnetic field. The features are well reproduced by micromagnetic simulations presented in Figures 4k–n.

## 3 Conclusions

Our observations reveal that a metastable antiskyrmion lattice can be created by quenching the thermodynamically stable phase near *T*_C_. Such a quenching process allows the antiskyrmions to persist in a wide *T*‐*µ_0_H* window, which importantly includes RT and zero magnetic field. On the other hand, by tuning the magnitude and direction of the external magnetic field, the metastable antiskyrmions can be transformed into skyrmions or non‐topological bubbles.

Moreover, we have studied the metastable antiskyrmion lattice in micron‐sized samples with various geometries. Besides forming the desired geometry, defining such small‐area confinement also improves the uniformity of sample thickness and reduces strain, as evidenced by the homogeneous magnetic contrast in the L‐TEM images. As a result, the geometry effect becomes a primary factor affecting the antiskyrmion lattice. A perfect square antiskyrmion lattice is promoted in a square‐shaped sample because the square edges are well aligned with the two orthogonal ***q*‐**vectors of the anisotropic helices. The square lattice of antiskyrmions deforms when the sample edges are not parallel to the ***q‐*** vectors. The lattice also distorts when some antiskyrmions transform into skyrmions and/or non‐topological bubbles, possibly owing to the interaction among various magnetic objects that have different symmetries. Particularly, antiskyrmions near the sample edge tend to deform and transform into non‐topological bubbles. Phenomenologically, the geometrical confinement accelerates the annihilation of magnetic textures in the confined sample with a lower saturation field, as compared to that in a thin plate with a much wider area,^[^ ^21^ ^]^ perhaps due to the demagnetization effect.

In summary, we have demonstrated a quenching procedure can yield metastable zero‐field antiskyrmions in Fe_1.9_Ni_0.9_Pd_0.2_P at and above room temperature. The transitions among topological and non‐topological spin textures as functions of temperature and external magnetic field were systematically characterized, and the formation and control of confined antiskyrmions were revealed. The impact of confining geometric structures on antiskyrmion and skyrmion lattices indicates the importance of inter‐particle and particle‐edge interactions. Our results of controlling the zero‐field antiskyrmion stability, the topological transformation, and the lattice form in confining geometry would provide useful information for manipulating antiskyrmions/skyrmions in future applications.

## 4 Experimental Section

### Sample Preparation

The single crystal of Fe_1.9_Ni_0.9_Pd_0.2_P was grown by self‐flux method.^[^ ^21^ ^]^ The crystalline structure and phase purity were confirmed using X‐ray diffraction. L‐TEM samples were cut from bulk crystals using a focused ion beam (FIB) system equipped with a gallium ion gun (NB‐5000, Hitachi, Japan). Various planar geometries were prepared by segmenting selected areas of the sample with tungsten that was deposited using the FIB system (Figure S1). The L‐TEM sample was mounted on a commercial micro‐electromechanical‐system‐based E‐chip (Protochips, USA), which allowed to rapidly heat or cool the sample.^[^ ^30^ ^]^ The sample orientation was checked using selected‐area electron diffraction in a JEM‐2100F microscope (JEOL, Japan).

### L‐TEM Observations

L‐TEM measurements were performed using a JEM‐2100F microscope (JEOL, Japan) equipped with a double‐tilt heating holder (Fusion Select, Protochips, USA). The external magnetic field applied perpendicular to the thin plate was controlled by changing the objective lens current. An in‐plane magnetic field was induced by tilting the sample. Magnetic induction field maps were derived from the under‐ and over‐focus L‐TEM images using the software package QPt (HREM Co., Japan) based on the transport‐of‐intensity equation.^[^ ^41^ ^]^

### Micromagnetic Simulations

Micromagnetic simulations were performed using the open‐source package Mumax3^[^ ^40^ ^]^ in which the authors have incorporated the anisotropic DMI. Material parameters were taken from previously reported experiments at RT as follows^[^ ^21^ ^]^: exchange stiffness *A* = 8.1 pJ m^–1^, uniaxial anisotropy constant along the [001] axis *K_u_* = 31 kJ m^–3^, saturation magnetization *M_s_* = 417 kA m^–1^ which relates to the demagnetization field term, and anisotropic DMI constant *D_x_ = −D_y_* = 0.2 mJ m^–2^. A 2560 × 2560 × 160 nm cluster containing 512 × 512 × 8 cells was simulated. A square, circular, or triangular geometry was set inside the cluster that matched the experimental geometry.

## Conflict of Interest

The authors declare no conflict of interest.

## Authors Contribution

L.C.P., Y. Taguchi, Y. Tokura, and X.Z.Y. jointly conceived the project. L.C.P. prepared the Fe_1.9_Ni_0.9_Pd_0.2_P TEM samples, performed and analyzed L‐TEM experiments, and wrote the manuscript with X.Z.Y. K.V.I. performed micromagnetic simulations. K.K. synthesized the Fe_1.9_Ni_0.9_Pd_0.2_P crystal with Y. Taguchi. All authors discussed the data and contributed to the manuscript.

## Supporting information

Supporting Information

## Data Availability

The data that support the findings of this study are available from the corresponding author upon reasonable request.
