# Supplementary material for: Formation and Control of Zero‐Field Antiskyrmions in Confining Geometries
Source: Adv Sci (Weinh). 2022 Aug 17;9(28):2202950. doi: 10.1002/advs.202202950 (PMC9534945; doi:10.1002/advs.202202950)
Supplement: Supplementary file 1 — Supporting Information [file ADVS-9-2202950-s001.pdf]

ADVANCED SCIENCE
Open Access

Supporting Information

for *Adv. Sci.*, DOI 10.1002/advs.202202950

Formation and Control of Zero-Field Antiskyrmions in Confining Geometries

Licong Peng, Konstantin V. Iakoubovskii, Kosuke Karube, Yasujiro Taguchi, Yoshinori Tokura and Xiuzhen Yu**

Supporting Information

Formation and control of zero-field antiskyrmions in confining geometries

Licong Peng, Konstantin V. Iakoubovskii, Kosuke Karube, Yasujiro Taguchi, Yoshinori Tokura, and Xiuzhen Yu**

*Corresponding authors, e-mail: licong.peng@riken.jp; yu_x@riken.jp.

1. Fabrication of $Fe_{1.9}Ni_{0.9}Pd_{0.2}P$ thin plate with confined geometries.

To confine magnetic skyrmions and antiskyrmions in various geometries, we fabricated $Fe_{1.9}Ni_{0.9}Pd_{0.2}P$ thin plates in a multistep procedure using a focused ion beam (FIB) system (NB5000, Hitachi). In Fig. S1, we outline the fabrication step where we defined square and circular geometries. First, we fix a $Fe_{1.9}Ni_{0.9}Pd_{0.2}P$ thin plate with a dimension of 25 μm × 5 μm × 5 μm on a Mo mesh (Fig. S1a). We then mill out a trench with a desired lateral shape and a half-depth thickness (~2.5 μm) (Fig. S1b), and fill the trench via tungsten deposition (Fig. S1c). Finally, we remove the unfilled layer from the back side of the sample (Fig. S1d), and thin and clean both sides of the sample to make it transparent to the electron beam (Fig. S1e). Figure S1f shows a schematic of square-shape $Fe_{1.9}Ni_{0.9}Pd_{0.2}P$ sample confined by amorphous W.

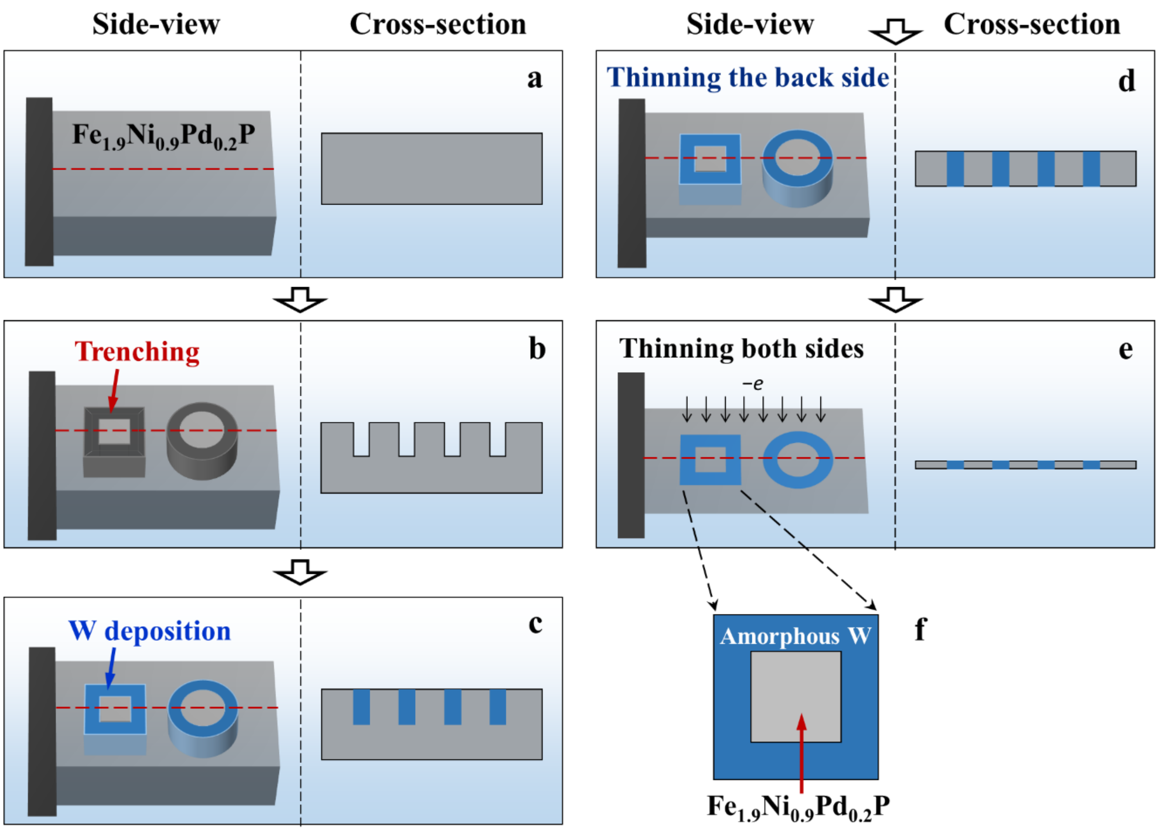

Figure S1. Schematic of sample fabrication. (a-e) Oblique views (left panels) and cross-sections (right panels) taken along the red dashed lines: (a) $Fe_{1.9}Ni_{0.9}Pd_{0.2}P$ plate fixed on a Mo mesh, (b) trenched geometries with square and circle shapes, (c) tungsten W deposition (presented in blue) within the trenched areas, (d) backside thinning, and (e) double-side thinning and cleaning of the sample. (f) Enlarged schematic from (e) showing the square-shape $Fe_{1.9}Ni_{0.9}Pd_{0.2}P$ sample confined by amorphous W.

2. Thermodynamically stable phase in the square-geometry $Fe_{1.9}Ni_{0.9}Pd_{0.2}P$.

L-TEM observations (Figs. S2a-c) demonstrate that sparse magnetic textures form from the helical stripes at 350 K as the field is increased. At 433 K, near T_C, the density of magnetic textures increases, and abundant magnetic textures are observed at 50 mT (Figs. S2d-e). Note here that the obtained magnetic contrast near T_C (Fig. S2e) is too weak to clearly distinguish the magnetic textures (antiskyrmions,

skyrmions, or non-topological bubbles). Hence, we count all magnetic textures to define the density for the thermodynamically stable phase.

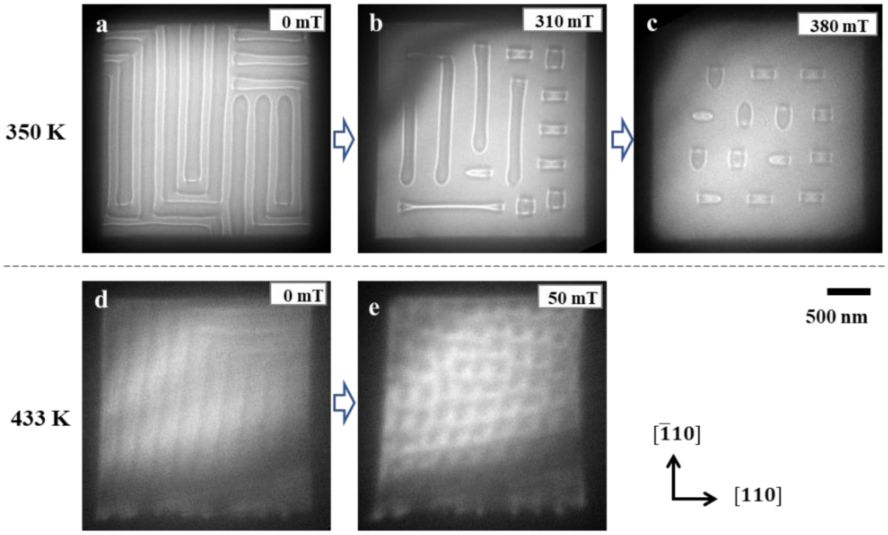

Figure S2. (a-e) L-TEM images of magnetic textures with increasing the magnetic field (after zero-field cooling) at (a-c) 350 K, and (d-e) 433 K.

3. Effect of cooling field and rate on the metastable antiskyrmion lattice.

The formation of metastable antiskyrmion lattice and the density of antiskyrmions strongly depend on the magnitude of the cooling field. This is a consequence of the small size of the thermodynamically stable T-μ_0H phase near T_C (Fig. S2). Figure S3 shows L-TEM images recorded after FC at different magnetic fields. Antiskyrmions coexist with helical stripes in the L-TEM images recorded at 15 mT (Fig. S3a), 80 mT (Fig. S3c) and 150 mT (Fig. S3d). The number of antiskyrmions is relatively high at 30 mT (Fig. S3b), but the antiskyrmion lattice is slightly distorted. The highest density of antiskyrmions and a well-ordered square lattice are obtained at an optimum field of 52 mT, as shown in Fig. 1e in the main text.

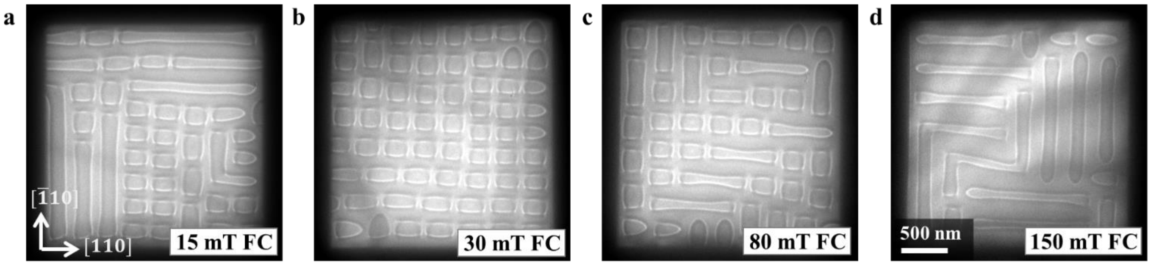

Figure S3. L-TEM images obtained after field cooling (FC) to room temperature at magnetic fields of (a) 15 mT, (b) 30 mT, (c) 80 mT, and (d) 150 mT.

Figure S4 shows L-TEM images of metastable antiskyrmion lattices obtained at various cooling rates. The density of antiskyrmions remains nearly unchanged in the studied range of 0.1-1000 K s^{-1}.

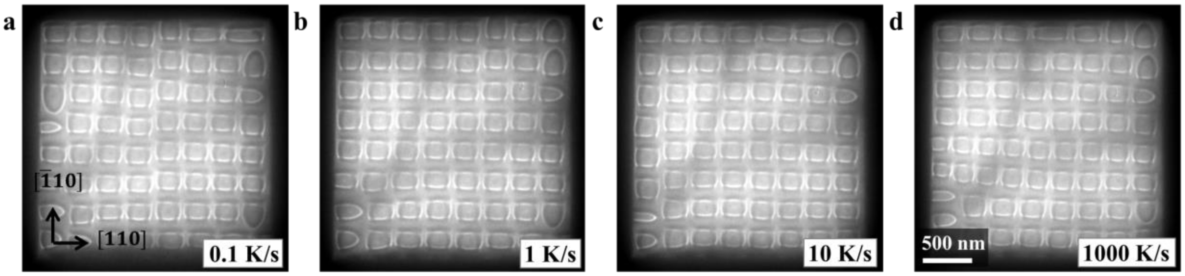

Figure S4. L-TEM images of antiskyrmion lattice observed at 52 mT for cooling rates of (a) 0.1 K s^{-1}, (b) 1 K s^{-1}, (c) 10 K s^{-1}, and (d) 1000 K s^{-1}.

4. Evolution of skyrmions with magnetic field.

Figure S5 shows the magnetic induction field map of the skyrmion lattice presented in Fig. 2g in the main text. The elliptical skyrmions with dark and white L-TEM contrast (Fig. S5a) exhibit two kinds of helicities, revealed by the corresponding magnetic induction field maps (Figs. S5b-c).

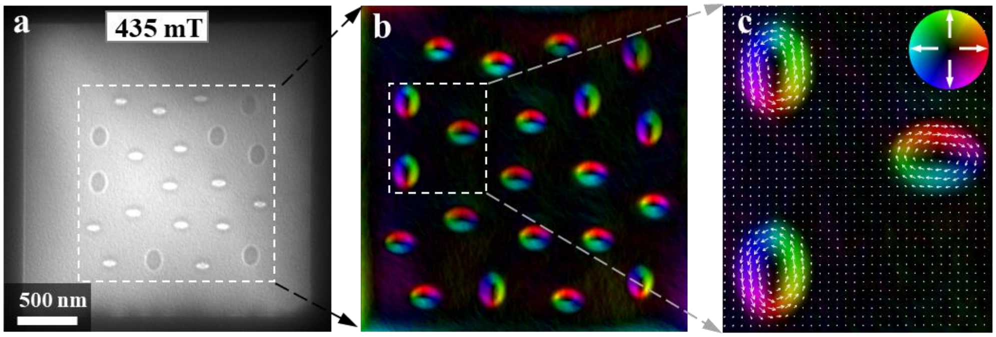

Figure S5. Magnetic induction field map of skyrmions. (a) L-TEM image showing the elliptical skyrmions with dark and white contrast at 435 mT at 295 K, and (b, c) corresponding magnetic induction field maps for the marked areas in (a, b). The vector direction is indicated by the color wheel (inset in c).

The number of skyrmions n_{sky} can be controlled by gradually increasing the magnetic field (Fig. S6). The skyrmions gather in the center of the defined geometry and form a (nearly) regular polygon, such as a square for $n_{sky} = 13$ (Fig. S6a), 9 (Fig. S6b), and 4 (Fig. S6e); a hexagon for $n_{sky} = 7$ (Fig. S6c), a pentagon for $n_{sky} = 6$ (Fig. S6d), and an equilateral triangle for $n_{sky} = 3$ (Fig. S6f). A pair of skyrmions and a single skyrmion are also observed (Figs. S6g-h).

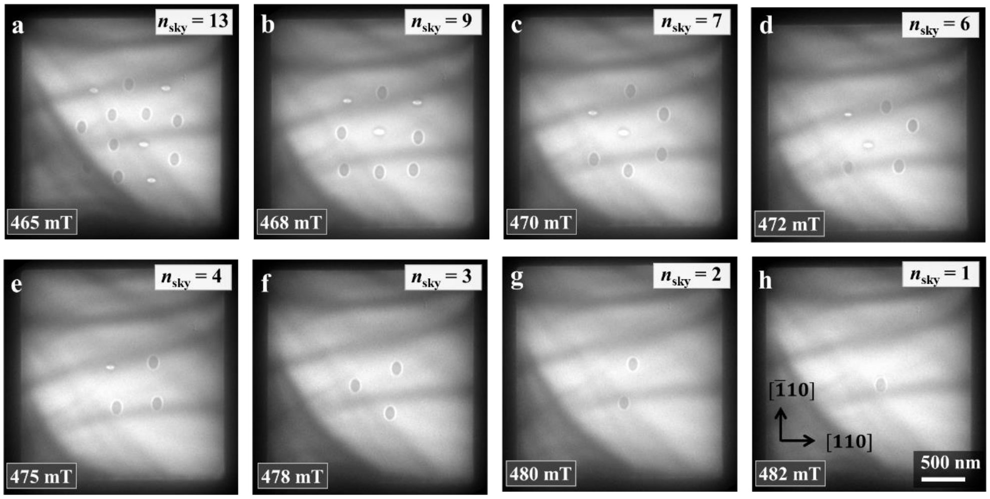

Fig. S6. (a-h) L-TEM images showing the collapse of skyrmions with increasing magnetic field.

5. Collapse of metastable antiskyrmions in oblique field

In Fig. S7, we present complementary L-TEM images for the transformation of metastable antiskyrmions in the oblique field. The zero-tilt L-TEM image (Fig. S7**a**) is similar to the Fig. 3**b** in the main text and shows the reproducibility of metastable square antiskyrmion lattice at 200 mT. Upon a −4° tilt, the square antiskyrmions transform into a mixture of trapezoid antiskyrmions and non-topological bubbles due to the in-plane magnetic field component $\mu_0 H_{||}$ (Fig. S7**b**). Upon the tilt to −8°, antiskyrmions change into a triangular lattice of non-topological bubbles (Fig. S7**c**).

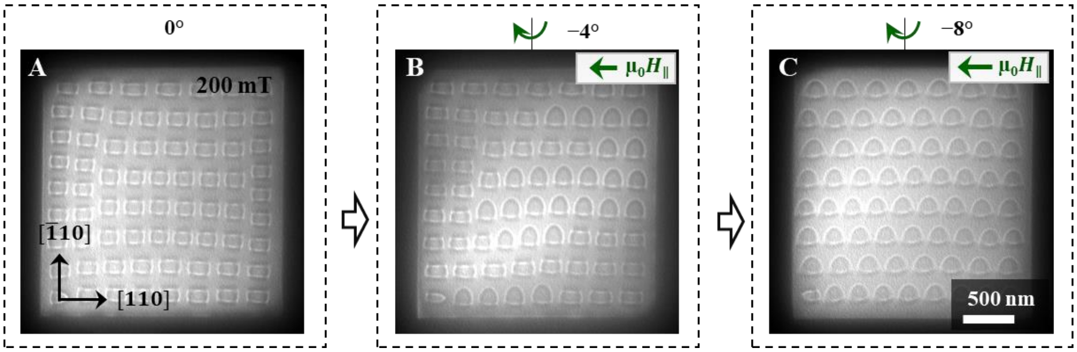

Figure S7. Effect of oblique fields on metastable antiskyrmions. (a-c) L-TEM images of (a) a square antiskyrmion lattice at 0°, (b) a mixture of trapezoid antiskyrmions and non-topological bubbles at −4°, and (c) a triangular lattice of non-topological bubbles at −8°.

6. Helical stripes in various geometries.

Figures S8a-c show L-TEM images of helical stripes at 0 mT in the rotated square, circular, and triangular geometries, respectively, revealing that the direction of ***q***-vectors of the helical stripes hardly depends on geometry. The ***q***-vectors are determined by the intrinsic material property, namely the directions of the anisotropic DMI vectors, which are aligned to the [110] and [$\bar{1}$10] axes. The experimental observations are supported by micromagnetic simulations (Figs. S8d-g).

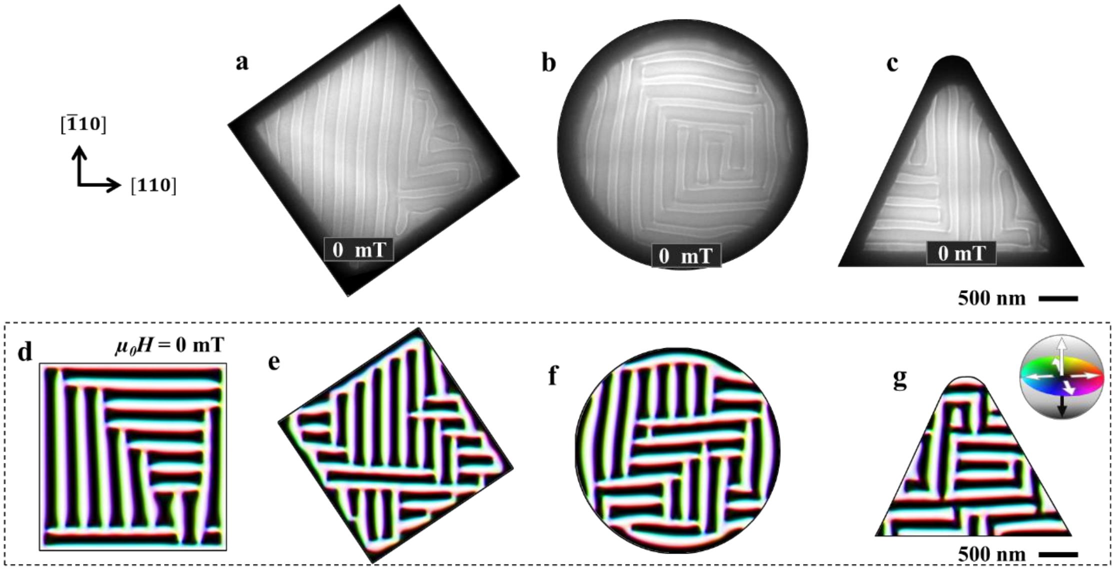

Figure S8. L-TEM images (a-c) and micromagnetic simulations (d-g) of helical stripes in various geometries at 0 mT.
